# Two-Hour Postprandial Lipoprotein Particle Concentration Differs Between Lean and Obese Individuals

**DOI:** 10.3389/fphys.2019.00856

**Published:** 2019-07-16

**Authors:** Ehsan Parvaresh Rizi, Sonia Baig, Tze Ping Loh, Sue-Anne Toh, Chin Meng Khoo, E. Shyong Tai

**Affiliations:** ^1^Department of Medicine, Yong Loo Lin School of Medicine, National University of Singapore, Singapore, Singapore; ^2^Department of Medicine, National University Health System, Singapore, Singapore; ^3^Department of Laboratory Medicine, National University Health System, Singapore, Singapore; ^4^Duke-National University of Singapore Medical School, Singapore, Singapore; ^5^Perelman School of Medicine, University of Pennsylvania, Philadelphia, PA, United States

**Keywords:** obesity, meal challenge, postprandial 2-h, macronutrients, lipoproteins

## Abstract

The concentrations of lipoprotein particles [high-density lipoproteins (HDLs), low-density lipoproteins (LDLs), very-low-density lipoproteins (VLDLs), and chylomicrons] are associated with the risk of cardiovascular diseases. Most studies have examined these associations in the fasting state. Previous studies have shown lipoprotein particle concentration change following meal, and these changes are different in individuals with obesity. In this study, we aimed to assess whether various meal compositions lead to adverse short-term (2-h) postprandial lipoproteinemia in obese insulin resistant (obese-IR) subjects as compared to lean insulin sensitive (lean-IS) subjects. In a randomized crossover trial, nine lean-IS and nine obese-IR Chinese men aged 22–35 years were challenged with isoenergetic and isovolumic meals rich in protein (HP), fat (HF), or carbohydrate (HC). Plasma samples were collected after a 10-h fast, as well as 1-h and 2-h post-meal and analyzed using nuclear magnetic resonance. Plasma concentration of large VLDLs and chylomicron particles was higher and increased more after all meals in obese-IR compared to lean-IS subjects. The HP meal decreased small LDL particle concentration in obese-IR subjects, and increased small HDL particle concentration in all subjects. The HF meal led to a decrease in small HDL concentration in all subjects. In conclusion, obese-IR subjects revealed a detrimental response to meal challenges even as early as 2-h after meal intake.

## Introduction

Plasma lipoproteins are heterogeneous, comprising particles of differing size and density. Very-low-density lipoproteins (VLDLs), low-density lipoproteins (LDLs), and high-density lipoproteins (HDLs) each comprise different sub-fractions and individually have an influence on cardiovascular disease (CVD) (Kwiterovich, 2002; Superko et al., 2012; Nikolic et al., 2013).

Most studies evaluating the association between lipoprotein subfraction and risk of CVD have been conducted in the fasting state (Garvey et al., 2003; Otvos et al., 2006; El Harchaoui et al., 2007). Previous studies have demonstrated that lipoprotein particle concentrations change after a meal (Wojczynski et al., 2011; Shah et al., 2017; Shah et al., 2018). Therefore, as people in industrialized societies spend a higher proportion of time in the postprandial state, postprandial lipoprotein particle concentrations might be a more relevant predictor of cardiovascular risk. A limited number of studies have been conducted following a high-fat (HF) mixed meal containing as much as 68.5% (Syvanne et al., 1997) to 83% of fat (Wojczynski et al., 2011) to assess postprandial lipoprotein particle concentration. However, this amount of fat is far higher than the recommended dietary intake (U. S. Department of Health and Human Services and U. S. Department of Agriculture, 2015), and assessment was between 6 and 8-h post-meal intake to elucidate peak levels of triglyceride-rich lipoproteins. The effect of a high-protein (HP) meal in a shorter timeframe of 3-h has been evaluated only by Shah et al. (2017, 2018) in lean and obese/overweight subjects. In clinical practice, there is a demand for reliable and practical assessment tools to investigate postprandial lipoproteinemia as a risk marker for CVD (Sciarrillo et al., 2019). In this study, we aimed to determine if a meal rich in carbohydrates, fat, or protein differentially alters the postprandial lipoprotein particle concentration as early as 2-h after meal challenge using a commercially available nuclear magnetic resonance (NMR) method. As a secondary aim, we sought to determine whether these effects differed between lean insulin-sensitive (lean-IS) and obese insulin-resistant (obese-IR) subjects.

## Materials and Methods

### Study Design

This study was carried out in accordance with the recommendations of Singapore’s National Healthcare Group Domain Specific Review Board with written informed consent from all subjects. All subjects gave written informed consent in accordance with the Declaration of Helsinki and the Singapore Good Clinical Practice guideline. The protocol was approved by the Singapore’s National Healthcare Group Domain Specific Review Board (DSRB Ref. No. C/2013/00902). The study design and meal tests for this study have been reported (Baig et al., 2016; Parvaresh Rizi et al., 2016). Eligible healthy volunteers participated in three crossover isoenergetic (≈600 kcal) liquid mixed meal tolerance tests with different macronutrient composition [rich in fat (HF), carbohydrate (HC), or protein (HP)], with 7 days washout in between each. Each meal was isovolumic (≈400 ml) and more than 50% of energy in HF, HC, and HP meals was derived from fat [with a 1:1:1 ratio of saturated, monounsaturated, and polyunsaturated fatty acids], carbohydrates, or protein, respectively (Supplementary Table 1). Order of the given test meals was randomly assigned to each subject over the study period. Key exclusion criteria included daily alcohol consumption >3 units, high level of physical activity (>5-h per week), and changes in body weight ≥5% within the preceding 3 months. Subjects were asked to keep their lifestyle unchanged during the study period (21 days).

All subjects were asked to have a light snack the night preceding test meal administration and to fast for 10-h before study procedures. The morning, an intravenous catheter was inserted in an antecubital vein, and subjects were given a 600-kcal liquid mixed meal to drink over 5 min. Fasting (*t* = 0 min) and postprandial (60 and 120 min) venous blood samples were collected through the inserted intravenous cannula for the determination of lipoprotein particle size and concentration using NMR. Blood samples were freshly centrifuged and serum was frozen and stored in Eppendorf Safe-Lock tubes at −80°C until the tests were performed at LipoScience^®^ Inc. Laboratories.

### Participants

All subjects provided written consent before recruitment into the study. Insulin resistance was defined based on the homeostasis model assessment for insulin resistance (HOMA-IR). Nine lean-IS (20 ≤ BMI < 23 kg/m^2^ and HOMA-IR < 1.2) and nine obese-IR (27.5 ≤ BMI < 35 kg/m^2^ and HOMA-IR ≥ 2.5) Chinese men aged 21–40 years were recruited. Inclusion and exclusion criteria, demographic data, and baseline plasma biochemical analysis of this cohort have previously been published (Baig et al., 2016; Parvaresh Rizi et al., 2016).

**Table 1:** Baseline subjects’ demographic and lipoprotein particle data.

	Lean-IS	Obese-IR	*P*	Adjusted *P^∗^*
Age (years)	23.2 ± 0.2	28.6 ± 1.4	0.002	
BMI (kg/m^2^)	22.0 ± 0.2	30.1 ± 0.7	<0.001	<0.001
Waist Circumference (cm)	79.9 ± 0.5	100.8 ± 1.0	<0.001	<0.001
HOMA-IR	0.83 ± 0.10	4.34 ± 0.41	<0.001	<0.001
**VLDL and CM Particle Concentration (nmol/L)**				
Total	58.50 ± 5.08	44.60 ± 7.19	0.136	0.112
Large	1.78 ± 0.37	6.19 ± 0.90	0.001	0.003
Medium	1.48 ± 0.83	3.83 ± 1.20	0.130	0.761
Small	55.23 ± 5.35	34.58 ± 7.79	0.046	0.062
**LDL Particle Concentration (nmol/L)**				
Total	702.63 ± 60.67	1161.15 ± 92.57	0.001	0.001
Large	218.33 ± 52.79	161.70 ± 59.97	0.489	0.588
Small	265.41 ± 49.95	854.96 ± 105.88	0.001	0.001
**IDL Particle Concentration (nmol/L)**				
IDL	218.85 ± 15.13	144.44 ± 19.01	0.033	0.025
**HDL Particle Concentration (μmol/L)**				
Total	30.04 ± 1.23	30.17 ± 1.58	0.949	0.055
Large	10.25 ± 0.73	5.64 ± 0.66	0.001	0.001
Medium	14.42 ± 1.02	12.13 ± 1.71	0.270	0.025
Small	5.38 ± 0.48	12.39 ± 2.15	0.006	0.007
**Mean Particle Size (nm)**				
VLDL	40.58 ± 1.65	56.09 ± 3.88	0.002	0.015
LDL	20.60 ± 0.16	19.99 ± 0.11	0.006	0.045
HDL	10.06 ± 0.08	9.21 ± 0.08	0.001	0.001
**NMR Calculated Concentration (mg/dl)**				
Total Triglyceride	61.18 ± 5.25	125.70 ± 17.39	0.003	0.088
Total VLDL and CM Triglyceride	61.18 ± 3.28	97.93 ± 10.53	0.004	0.192
Total HDL Cholesterol	58.52 ± 2.37	39.82 ± 3.18	0.001	0.001

*Data are presented as the mean ± SEM; n = 9 for each group. P < 0.05 is considered significant. ^∗^P value adjusted for age.*

**Table 2:** Time course analysis of lipoprotein particle concentration in lean-IS and obese-IR subjects after consuming three isocaloric liquid mixed meals.

		Main effect	Interaction effect		
		Time	Time × Meal	Time × Group	Time × Group × Meal
VLDL and CM	Total	0.002	0.152	0.688	0.980
	Large	0.036	0.433	0.006	0.375
	Medium	0.851	0.188	0.930	0.753
	Small	0.002	0.131	0.764	0.949
LDL	Total	0.062	0.001	0.474	0.686
	Large	0.302	0.426	0.385	0.569
	Small	0.134	0.005	0.643	0.398
IDL	IDL	0.104	0.606	0.315	0.908
HDL	Total	0.723	0.183	0.146	0.456
	Large	0.318	0.100	0.434	0.420
	Medium	0.278	0.121	0.311	0.582
	Small	0.153	0.039	0.264	0.925

*P values for main and interaction effects using repeated-measures ANOVA, adjusted for age.*

### Plasma Lipoprotein Analysis by NMR

The NMR LipoProfile^®^ test (performed by LipoScience^®^ Inc., Raleigh, NC, United States) used a Vantera^®^ Clinical Analyser, an automated proton NMR spectrometer, to determine LDL, HDL, and VLDL particle size and concentrations. This test involves measurement of the 400-MHz proton NMR spectrum of a plasma sample, deconvolution of the composite signal at ∼0.8 ppm to produce the signal amplitudes of the lipoprotein subclasses that contribute to the composite plasma signal, and conversion of these subclass signal amplitudes to lipoprotein subclass concentrations. The ∼0.8 ppm plasma NMR signal arises from the methyl group protons of the lipids carried in the LDL, HDL, and VLDL subclasses of varying diameter. The NMR signals from the various lipoprotein subclasses have unique and distinctive frequencies and line shapes, each of which is accounted for in the deconvolution analysis model. Each subclass signal amplitude is proportional to the number of subclass particles emitting the signal, which enables subclass particle concentrations to be calculated from the subclass signal amplitudes derived from the spectral deconvolution analysis. Plasma concentrations of LDL, VLDL, and chylomicron (CM) subclasses were reported in nmol/L, and those of HDL were reported in μmol/L. The average size of different lipoprotein particles in nanometers (nm) is as follows: intermediate density lipoprotein (IDL) = 23–27; large LDL = 21.2–23; small LDL = 18–21.2; large HDL = 8.8–13; medium HDL = 8.2–8.8; small HDL = 7.3–8.2; large VLDL = 60–200; medium VLDL = 35–60; and small VLDL = 27–35. By employing conversion factors that assume the various lipoprotein subclass particles have cholesterol and triglyceride contents characteristic of normolipidemic individuals, HDL cholesterol and triglyceride concentrations were also derived (Otvos, 2002). LDL measured by the NMR LipoProfile^®^ test has been shown to be a determinant of CVD risk in two previously reported prospective case-control studies (Otvos et al., 2006; El Harchaoui et al., 2007).

**FIGURE 1:**
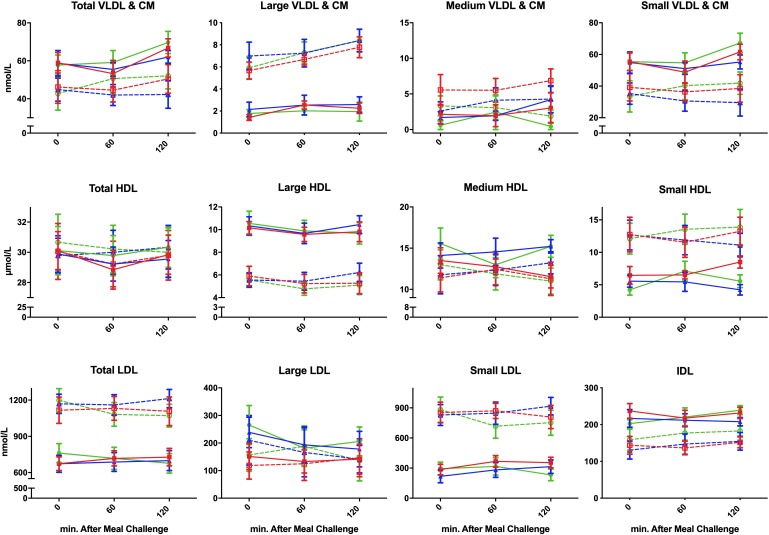
Postprandial concentration of different plasma lipoproteins following ingestion of three different isocaloric liquid mixed meals in nine lean insulin-sensitive subjects following high-protein (—–, 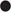), high-fat (—–, 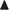), or high-carbohydrate (—–, 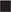) isocaloric liquid mixed meals ingestion, and nine obese insulin-resistant following high-protein (----, 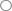), high-fat (----, Δ), or high-carbohydrate (----, 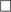) isocaloric liquid mixed meals ingestion. Values are means ± SEM.

### Statistical Analysis

This study is a *post hoc* analysis of samples from a study designed to investigate inflammatory responses after three different meal challenges (Parvaresh Rizi et al., 2016). All statistical analyses were performed using SPSS version 23.0 for Windows (SPSS Inc., Chicago, IL, United States). All values are presented as means ± SEM. Student’s *t* test was used to test the continuous variables between groups. The time course of each postprandial plasma lipoprotein particle concentrations was analyzed by repeated-measures ANOVA and age was adjusted as a covariate. Differences of postprandial response between meals as well as groups were assessed *via* time × meal, time × group, and time × meal × group interaction tests. A *P* value of < 0.05 was considered statistically significant.

## Results

### Baseline Demographic Data and Fasting Lipoprotein Plasma Level (Table 1)

Subjects’ demographic data and mean fasting plasma concentration of different lipoproteins are presented in Table 1. Obese-IR subjects were older (age range: lean-IS: 23–25, obese-IR: 22–35) and had higher body mass index (BMI), waist circumference, and HOMA-IR.

**FIGURE 2:**
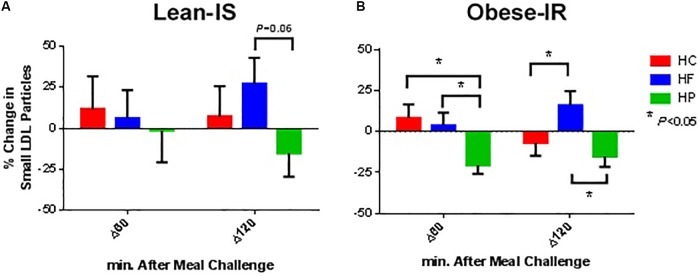
Percentage change from baseline for plasma small LDL particle concentration at 60 and 120 min following ingestion of three different isocaloric mixed meals. High-protein (green), high-fat (blue), or high-carbohydrate (red) liquid mixed meals in panel **(A)** nine lean insulin-sensitive and **(B)** nine obese insulin-resistant subjects. Values are means ± SEM.

**FIGURE 3:**
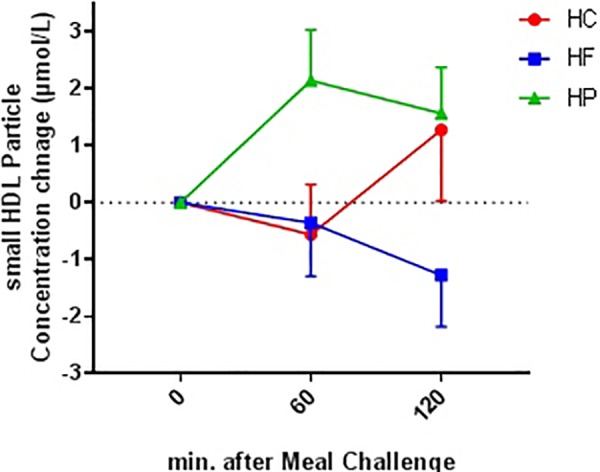
Absolute change from baseline for plasma small HDL particle concentration at 60 and 120 min following ingestion of three different isocaloric mixed meal. High-protein (green), high-fat (blue), or high- carbohydrate (red) liquid mixed meals. Values are means ± SEM. *N* = 18. *P*_time×meal_ interaction = 0.043.

The fasting plasma concentration of different lipoproteins is the average of three fasting samples. The total number of VLDL and CM particles was similar among groups, but obese-IR subjects had higher concentrations of large VLDL and lower concentrations of small VLDL particles (statistically non-significant after age adjustment). LDL particle concentration was higher in obese-IR subjects due to higher concentrations of small LDL particles. Although total HDL particle concentrations did not differ between groups, obese-IR subjects had higher concentration of small HDL particles and lower concentration of large HDL particles. These differences led to larger VLDL and smaller LDL and HDL particle size in obese-IR subjects. Calculated triglyceride was higher, and HDL-cholesterol was lower in obese-IR subjects (statistically non-significant after age adjustment).

### Postprandial Lipoprotein Particle Concentration (Table 2 and Figure 1)

#### VLDL and CM

The concentration of total, large, and small particles increased after consumption of a meal (*P*_time_ < 0.05). There was no difference in the change in postprandial particle concentration between the different meals (*P*_time×meal_ interaction > 0.05). However, obese-IR subjects showed a greater increase in concentration of large particles after all three meals compared to lean-IS subjects (*P*_time × group_ interaction < 0.001).

#### LDL

Low-density lipoprotein particle concentration was essentially unchanged after a meal, except for the HP meal where a reduction in the concentration of total and small particle was observed (*P*_time×meal_ interaction < 0.01 for both total and small LDL particles). This was more prominent in obese-IR subjects, but the interaction term time × meal × group was not statistically significant (Figure 2).

#### IDL

There was no change in concentration of IDL particle from the fasting to the postprandial state, nor was there any difference in the change from fasting to postprandial state between different meals or groups.

#### HDL

Concentration of total and large particles decreased after consumption of a meal (*P*_time_ < 0.001); however, no difference was observed after adjustment for age (Table 2). There was no difference in the change in postprandial particle concentration between obese-IR and lean-IS subjects (*P*_time×group_ interaction > 0.05). HP meal induced an increase whereas HF meal resulted in a decrease in concentration of small particles (*P*_time × meal_ interaction < 0.05) (Figure 3).

## Discussion

This study showed that postprandial increase in large VLDL and CM particles was more pronounced in obese-IR than in lean-IS subjects, which was evident within 2-h of meal ingestion. We also examined the effect of different macronutrient composition of test meals and found that ingestion of an HP meal was associated with a reduction in small LDL particle concentration, which occurred in tandem with an increase in small HDL particle concentration. In contrast, an HF meal decreased small HDL particle concentration but had no specific impact on LDL particle concentration.

Previous studies have shown that a HF meal (83% calorie from fat) results in a greater increase in large VLDL and CM particle concentration compared to other lipoprotein particles (Wojczynski et al., 2011). In our study, we did not detect any differences in the effect of different meal compositions on postprandial VLDL and CM particle concentration. Our finding in relation to greater increases in large VLDL and CM particle concentrations in obese-IR subjects after a HF meal is consistent with the existing literature (Cohn, 2006; Wojczynski et al., 2011). Here, we extend these findings by showing that this phenomenon also occurred following HP and HC meals in obese-IR subjects.

Small LDL particles originate from liver-secreted precursors (mainly triglyceride-rich lipoproteins such as large VLDL particles), and lipoprotein lipase (LPL), hepatic lipase (HL), and cholesteryl ester transfer protein (CETP) mediate this conversion process (Ivanova et al., 2017). CETP mediates exchange of cholesteryl ester and triglycerides between LDL and VLDL and/or HDL. HL plays a pivotal role in metabolism of these particles, acting on the core triglycerides and surface phospholipids. Moreover, LDL particles can be catabolized into smaller and denser particles through HL activity, which increases during the postprandial state (Diffenderfer and Schaefer, 2014). We showed that the macronutrient composition of the meal affects the postprandial plasma concentration of small LDL particles, which decreased after a HP meal but increased after a HF meal. There is no evidence that HL activity is altered after a HP meal, particularly in obese-IR subjects in whom we saw a greatest reduction in small LDL particle concentration after the HP meal. Although VLDL and HDL particles can serve as substrates to produce small LDL particles or lipid transfer between particles, we did not observe changes in VLDL and HDL particles that were specific to the HP meal. We do recognize that our relatively small sample size and short postprandial period may have limited our ability to detect these changes. Moreover, LDL particle size and density are affected by carbohydrate metabolism, and HC intake generates smaller LDL particles than a low carbohydrate diet (Gerber and Berneis, 2012). We have previously shown that a HP meal resulted in low postprandial blood glucose and high plasma insulin in these individuals (Parvaresh Rizi et al., 2016). Therefore, it is possible that altered carbohydrate metabolism after a HP meal resulted in altered production of LDL particles. Nevertheless, the underlying mechanism for this observation warrants further mechanistic research.

Plasma HDL concentration has shown to be inversely associated with risk of CVD. However, emerging findings demonstrate that such correlation is more defined by function of HDL particles rather than their plasma level (Pirillo et al., 2013). With regard to the postprandial changes in HDL particle concentration, limited evidence indicates a minimal (Wojczynski et al., 2011) to detrimental (De Bruin et al., 1991) effect of a HF meal on postprandial HDL particle concentration. To our understanding, Shah et al. (2017, 2018) are the only group who has compared the effects of HP and high-monounsaturated fat (HMF) meals on postprandial HDL concentration and particle number in lean (Shah et al., 2017) and overweight/obese (Shah et al., 2018) subjects, albeit in separate studies. In both lean and overweight/obese subjects, they showed that the HP meal compared to the HMF meal increased small-dense HDL particles. The study design, subjects’ characteristics, and composition of test meals used in these studies are different compared to our study. However, in agreement with Shah et al. (2018), we did not observe any differential postprandial response for HDL particle concentration between obese-IR and lean-IS subjects. In addition, in our study as well as theirs, intake of a HP meal was associated with an increase in small HDL particle concentration and intake of a HF meal was associated with decreased small HDL particle concentration. A lack of understanding of the underlying mechanism for similarities in these observations warrants further investigation; nevertheless, an increase in small HDL particle concentration following the HP meal might be due to decreased production of CETP.

This study offers no mechanistic findings and only presents observations in different subjects taking three different meals. Furthermore, the NMR method that we used in this study cannot distinguish between the two major triacylglycerol-rich lipoprotein groups, VLDL and CM.

In conclusion, we found an increase in postprandial large VLDL and CM particle concentrations in obese-IR compared to lean-IS subjects. Additionally, a HP meal decreased small LDL particle concentration and increased small HDL particle concentration.

## Ethics Statement

This study was carried out in accordance with the recommendations of Singapore’s National Healthcare Group Domain Specific Review Board with written informed consent from all subjects. All subjects gave written informed consent in accordance with the Declaration of Helsinki and the Singapore Good Clinical Practice guideline. The protocol was approved by the Singapore’s National Healthcare Group Domain Specific Review Board (DSRB Ref. No. C/2013/00902).

## Author Contributions

EPR, TL, S-AT, CK, and ET designed the research. EPR and SB conducted the research. EPR analyzed the data. EPR and ET had primary responsibility for the final content. All authors wrote the manuscript and read and approved the final manuscript.

## Conflict of Interest Statement

The authors declare that the research was conducted in the absence of any commercial or financial relationships that could be construed as a potential conflict of interest.
