# Supplementary material for: Two-Hour Postprandial Lipoprotein Particle Concentration Differs Between Lean and Obese Individuals
Source: Front Physiol. 2019 Jul 16;10:856. doi: 10.3389/fphys.2019.00856 (PMC6649689; doi:10.3389/fphys.2019.00856)
Supplement: Supplementary file 1 [file Table_1.DOCX]

**Supplementary Table 1.** Macronutrient composition of the 3 different liquid mixed meals

|  |  | **Amount**  **(g)** | **Energy**  **(Kcal)** | **Carb.**  **(g)** | **Protein**  **(g)** | **Fat**  **(g)** | **MUFA**  **(g)** | **PUFA**  **(g)** | **SFA**  **(g)** |
| --- | --- | --- | --- | --- | --- | --- | --- | --- | --- |
| **HF Meal** | *Ensure Plus^®^* | 260.0 | 366.60 | 51.69 | 13.00 | 11.70 | 2.85 | 6.90 | 1.48 |
|  | Butter | 11.0 | 78.87 | 0.01 | 0.09 | 8.80 | 2.31 | 0.33 | 5.61 |
|  | Diary Cream Heavy Whipping | 11.0 | 37.95 | 0.31 | 0.22 | 4.07 | 1.10 | 0.15 | 2.53 |
|  | Peanut Oil | 13.0 | 114.92 | 0.00 | 0.00 | 13.00 | 5.98 | 4.16 | 2.18 |
|  | Total |  | **598.34** | 52.00 | 13.31 | 37.57 | 12.24 | 11.54 | 11.81 |
|  | % composition |  |  |  |  |  | 32.6 | 30.7 | 31.4 |
|  | % Kcal |  |  | 34.8 | 8.9 | **56.5** |  |  |  |
| **HC Meal** | *Ensure Plus^®^* | 425.0 | 599.25 | 84.49 | 21.25 | 19.13 | 4.65 | 11.28 | 2.42 |
|  | Total |  | **599.25** | 84.49 | 21.25 | 19.13 | 4.65 | 11.28 | 2.42 |
|  | % composition |  |  |  |  |  | 24.3 | 59.0 | 12.7 |
|  | % Kcal |  |  | **56.4** | 14.2 | 28.7 |  |  |  |
| **HP Meal** | *Ensure Plus^®^* | 230.0 | 324.30 | 45.72 | 11.50 | 10.35 | 2.52 | 6.11 | 1.31 |
|  | *Beneprotein^®^* Powder | 77.0 | 274.89 | 0.00 | 65.45 | 0.00 | 0.00 | 0.00 | 0.00 |
|  | Total |  | **599.19** | 45.72 | 76.95 | 10.35 | 2.52 | 6.11 | 1.31 |
|  | % composition |  |  |  |  |  | 24.3 | 59.0 | 12.7 |
|  | % Kcal |  |  | 30.5 | **51.4** | 15.5 |  |  |  |

HC, high carbohydrate, HF, high fat, HP, high protein, MUFA, monounsaturated fatty acids, PUFA, polyunsaturated fatty acids, SFA, saturated fatty acids, *Ensure Plus^®^* (1g=1.41kcal, 0.05g protein, 0.045g fat, 0.1988g carbohydrate, 0.0057g SFA, 0.01095g MUFA, 0.02655g PUFA, 0g fibre) manufactured by *Abbott Nutrition* was used as a benchmark for HC meal; *Beneprotein^®^* (1g powder=3.57kcal, 0g fat, 0g carbohydrate, 0.85g protein, 5mg potassium, 5.7mg calcium, 2mg phosphorus, 0g fibre) is manufactured by *Nestlé Nutrition*.
